# Predictive Complexity of Quantum Subsystems

**DOI:** 10.3390/e26121065

**Published:** 2024-12-07

**Authors:** Curtis T. Asplund, Elisa Panciu

**Affiliations:** 1 Department of Physics & Astronomy, San José State University, One Washington Square, San José, CA 95192-0106, USA; 2 Department of Physics, University of Maryland, College Park, MD 20742-4111, USA

**Keywords:** entanglement entropy, predictive complexity, Heisenberg model, Lieb–Robinson bound, spin wave, local order parameter

## Abstract

We define predictive states and predictive complexity for quantum systems composed of distinct subsystems. This complexity is a generalization of entanglement entropy. It is inspired by the statistical or forecasting complexity of predictive state analysis of stochastic and complex systems theory but is intrinsically quantum. Predictive states of a subsystem are formed by equivalence classes of state vectors in the exterior Hilbert space that effectively predict the same future behavior of that subsystem for some time. As an illustrative example, we present calculations in the dynamics of an isotropic Heisenberg model spin chain and show that, in comparison to the entanglement entropy, the predictive complexity better signifies dynamically important events, such as magnon collisions. It can also serve as a local order parameter that can distinguish long and short range entanglement.

## 1. Introduction

Quantum systems of many particles are capable of an astonishing variety of complicated behaviors. Characterizing these behaviors in a useful way can be challenging. Building on the foundation of entanglement entropy, and incorporating ideas from complex systems theory, we define new quantities for such systems: predictive states and predictive complexity. We then apply them to a prototypical example, a Heisenberg model spin chain, and find that they provide an improved measure of some dynamical processes and they can serve as promising new tools for analyzing general quantum dynamical systems.

We are primarily guided by two lines of research. On one hand, entanglement entropy of lattice systems and field theories has been a highly successful tool in recent times, from the celebrated area law [1] to many applications in quantum dynamics [2,3]. On the other hand, we have the statistical or forecasting complexity of stochastic and complex systems [4,5,6], which has developed into a sophisticated formalism that has been applied to numerous systems [7,8,9].

We distinguish our definition of predictive complexity from the quantum Hamiltonian complexity [10,11], which classifies systems according to complexity classes, and so does not determine a definite complexity value. It is also distinct from the quantum computational or gate complexity [12,13], which is the basis for most work in high-energy physics and gauge/gravity (AdS/CFT) duality [14,15]. In particular, our construction is information-theoretic and has no dependence on a choice of gates or reference state.

Our definitions can be seen as quantum extensions of predictive state analyses of classical spin systems [16,17,18,19], cellular automata [20], various spatio-temporal dynamical systems [21,22], and of optimal quantum models of stochastic systems [23,24,25,26,27,28]. It is similar in spirit to using the statistical complexity to analyze the distribution of measurement outcomes of a quantum system [29,30,31], but we do not involve measurement distributions of any particular observable.

Several other quantities called “complexity” have been applied to quantum and spin systems [32,33,34,35,36,37]. These are defined differently to the complexity we define here, but relations between these would be interesting to investigate further.

## 2. Methods and Definitions

### 2.1. Predictive Equivalence

Consider a quantum system composed of a subsystem *A* and its complement or exterior *B*. We have in mind a finite set of interacting particles, spins, or qubits, with *A* consisting of a subset. Accordingly, we write the finite-dimensional Hilbert space H of this system as

(1) $$H=H_{A}⊗H_{B}\;.$$

For any density operator of such a system, one can form the reduced density operators using partial traces $\rho_{A}={\mathrm{tr}}_{B}\rho$ and $\rho_{B}={\mathrm{tr}}_{A}\rho$. In the case of a pure state, ρ=|ψ〉〈ψ|, the von Neumann entropies are equal and give the entanglement entropy between *A* and *B*:

(2) $$S_{A}=-\mathrm{tr}\left(\rho_{A}\log\rho_{A}\right)=-\mathrm{tr}\left(\rho_{B}\log\rho_{B}\right)=S_{B}\;.$$

From information theory (coding theorems), this entropy may be interpreted as the amount of information needed to describe the state of *A*, on average, using a suitable encoding of a basis of $H_{A}$. We may also interpret this as the amount of information about *B*, relevant to *A*, that has been lost due to tracing out *B*. This measures how much *A* is entangled with *B* and vice versa.

Grouping or coarse-graining outcomes of a stochastic process generally leads to a reduction in entropy since less precise information is needed to describe the outcomes. Suppose some states in $H_{B}$ were equivalent, in some sense, say, in terms of how they affect the evolution of $\rho_{A}$. Then, we would expect to need less information about what is going on in *B* to effectively predict the behavior in *A*. Below, we describe how to implement any such equivalence in terms of how it affects $\rho_{A}$ and the entropy. We focus on an equivalence defined by identical evolution of a subsystem for some time, and the consequently reduced entropy is thus a measure of the average minimal information needed to sufficiently predict the state of *A*, or observables in *A*, for that time. This is the rough idea for what we call the predictive complexity.

We are following the ideas of causal or predictive state analysis, sometimes also called computational mechanics [4,5,6]. In the context of classical and stochastic dynamical systems, this type of analysis defines an equivalence relation between states that yield equivalent probabilistic predictions for the system.

In our quantum context, let us call two states $|\phi_{1}\rangle ,|\phi_{2}\rangle \in H_{B}$, equivalent if and only if they lead to the same dynamics in *A*. More precisely, for a given $|\psi \rangle \in H_{A}$, we can form the states $|\Psi_{1}\rangle =|\psi \rangle |\phi_{1}\rangle ,|\Psi_{2}\rangle =|\psi \rangle |\phi_{2}\rangle \in H$ and the corresponding density operators $\rho_{1}=\left|\psi \rangle \right|\phi_{1}\rangle \langle \phi_{1}\left|\langle \psi \right|$ and $\rho_{2}=\left|\psi \rangle \right|\phi_{2}\rangle \langle \phi_{2}\left|\langle \psi \right|$. Let us write $\rho_{1}\left(t\right)$ and $\rho_{2}\left(t\right)$ for the time-evolved density operators, i.e., if $U\left(t\right)=U_{t}=e^{-iHt/\hbar }$ is the time-evolution operator for a time-independent, whole-system Hamiltonian *H*, $\rho_{1}\left(t\right)=U\left(t\right)\rho_{1}U^{†}\left(t\right)$. Then, we can define $|\phi_{1}\rangle$ and $|\phi_{2}\rangle$ to be equivalent if and only if

(3) $${\mathrm{tr}}_{B}\rho_{1}\left(t\right)={\mathrm{tr}}_{B}\rho_{2}\left(t\right)\;,$$

for all $|\psi \rangle \in H_{A}$ and for some interval of time, which we call the time horizon $t_{h}$.

Reflexivity, symmetry, and transitivity of the relation follow quickly from Equation (3), so it is an equivalence relation. This also comes from it being defined by the equality of the images of a function on $H_{B}$.

One can express Equation (3) in terms of relative entropy, or Kullback–Leibler divergence, defined for any two density operators ρ and σ to be S(ρ∥σ)≡tr(ρlogρ−ρlogσ). Since the relative entropy of two density operators is zero if and only if they are equal, Equation (3) becomes

(4) $$S\left({\mathrm{tr}}_{B}\rho_{1}\left(t\right)∥{\mathrm{tr}}_{B}\rho_{2}\left(t\right)\right)=0\;.$$

Equivalently, one could express this by saying the distance between the two density operators is zero for a given interval of time, under any metric on the space of density operators, such as the trace distance. In fact, all one needs to have well-defined equivalence classes is a partition of $H_{B}$, and this could come from a generalization of Equation (4), where the right-hand side is not strictly zero but just sufficiently close to zero so that it leads to sufficient clustering of states in $H_{B}$ to be partitioned (in a way that respects the linear and inner product structure, as we describe further below). We plan to investigate such generalizations in future work.

The relation clearly depends on the time horizon. In relativistic systems and in a case where *A* is taken to be a compact spatial region, this would translate into a spatial horizon distance $r_{h}=t_{h}/c$. By relativistic causality, we expect all states of $H_{B}$ that only differ in their configurations at distances from *A* greater than $r_{h}$ to be equivalent to each other. We have similar expectations for quantum systems obeying a Lieb–Robinson bound since this similarly limits the speed of propagation of information [38]. In that case, though, exponentially small corrections are allowed. This is where small corrections to the right-hand side of Equation (4) could be important. We are interested in the states in $H_{B}$ that can be partitioned into equivalence classes in terms of their effect on the subsystem *A*. We will explicitly see how this works in the case of the Heisenberg model below.

As we will see, the linear and inner product structure of H combined with the unitary evolution operator naturally gives rise to equivalence classes that can be thought of as parallel affine subspaces, akin to superselection sectors. They may also be interpreted as projective subspaces in projective Hilbert space, which can be a useful perspective since it quotients out un-normalized state vectors and we are ultimately interested in normalized states and density operators. (Projective Hilbert space, or ray space, is the space formed from a Hilbert space by taking the quotient by the relation |ψ〉∼z|ψ〉, where *z* is any non-zero complex number. It can be thought of as the space of physically distinct states in H [39,40,41]).

The predictive equivalence defined by Equation (3) is natural from the point of view of reduced density operators, but to define an equivalence relation with the desired linearity properties on the Hilbert space $H_{B}$, we need a further requirement on the dynamics of the equivalent states. To see this, let $|\phi_{1}\rangle ,|\phi_{2}\rangle \in H_{B}$ be normalized, orthogonal state vectors satisfying Equation (3). Then, let $|\phi_{3}\rangle =a_{1}|\phi_{1}\rangle +a_{2}|\phi_{2}\rangle \neq 0$ with $|a_{1}{|}^{2}+{\left|a_{2}\right|}^{2}=1$, i.e., an arbitrary normalized linear combination. We can use a Schmidt decomposition to write the evolution of the states $|\Psi_{1}\rangle =|\psi \rangle |\phi_{1}\rangle ,|\Psi_{2}\rangle =|\psi \rangle |\phi_{2}\rangle$ (in the Schrödinger picture of time evolution) as

(5) $$\begin{array}{cc} |\Psi_{1}\left(t\right)\rangle & =U_{t}|\Psi_{1}\rangle =c_{1}^{ij}\left(t\right)|\psi^{i}\rangle |\phi^{j}\rangle \\ |\Psi_{2}\left(t\right)\rangle & =U_{t}|\Psi_{2}\rangle =c_{2}^{ij}\left(t\right)|\psi^{i}\rangle |\phi^{j}\rangle \;, \end{array}$$

where *i* and *j* are summed over bases for $H_{A}$ and $H_{B}$, respectively. The evolution of $\left|\psi \rangle \right|\phi_{3}\rangle$ can then be written

(6) $$\begin{array}{c} U_{t}|\psi \rangle (a_{1}|\phi_{1}\rangle +a_{2}|\phi_{2}\rangle )=a_{1}c_{1}^{ij}\left(t\right)|\psi^{i}\rangle |\phi^{j}\rangle +a_{2}c_{2}^{ij}\left(t\right)|\psi^{i}\rangle |\phi^{j}\rangle \;. \end{array}$$

A short calculation shows that to ensure the property in Equation (3) holds for $\rho_{3}$, we need, for all *i* and *k*,

(7) $$\sum_{j}c_{1}^{ij}\left(t\right)c_{2}^{kj*}\left(t\right)=0\;.$$

This can be understood as requiring that $U_{t}$ maintain orthogonality between equivalent states in $H_{B}$, and it can also be rewritten

(8) $${\mathrm{tr}}_{B}|\Psi_{1}\left(t\right)\rangle \langle \Psi_{2}\left(t\right)|=0\;.$$

We have written this as a strict operator equation, but as with our discussion of Equation (3), all we need for our construction to work is for the operator $O_{A}:={\mathrm{tr}}_{B}|\Psi_{1}\left(t\right)\rangle \langle \Psi_{2}\left(t\right)|$ to be sufficiently small, in the sense of whatever metric one is using. For example, with respect to trace distance, Equation (8) would be replaced with a requirement that the trace norm ${∥O_{A}∥}_{1}=\mathrm{tr}\sqrt{O_{A}^{†}O_{A}}$ is small compared with the trace distance between any reduced density operators generated by inequivalent states, for times within the time horizon. Below, we argue that this holds for many systems and we numerically verify it for our main example system of a Heisenberg spin chain.

It is straightforward to show that Equation (8) holds when *A* and *B* are decoupled since then we may write $|\Psi_{i}\left(t\right)\rangle =|\psi \left(t\right)\rangle |\phi_{i}\left(t\right)\rangle$. Thus,

(9) $$\begin{array}{cc} \; & {\mathrm{tr}}_{B}|\Psi_{1}\left(t\right)\rangle \langle \Psi_{2}\left(t\right)|\\ \; & =\langle \phi^{i}\left||\psi \left(t\right)\rangle \right|\phi_{1}\left(t\right)\rangle \langle \psi \left(t\right)|\langle \phi_{2}\left(t\right)||\phi^{i}\rangle \\ \; & =\langle \phi_{2}\left(t\right)||\phi^{i}\rangle \langle \phi^{i}\left|\right|\phi_{1}\left(t\right)\rangle |\psi \left(t\right)\rangle \langle \psi \left(t\right)|\\ \; & =\langle \phi_{2}\left(t\right)|\phi_{1}\left(t\right)\rangle |\psi \left(t\right)\rangle \langle \psi \left(t\right)|\;, \end{array}$$

which equals zero since $|\phi_{1}\rangle$ and $|\phi_{2}\rangle$ were assumed to be orthogonal, and this is preserved if the dynamics in *A* and *B* are decoupled.

To see what this means in terms of the system Hamiltonian *H* that describes local interactions in a spatially extended system, let us assume that it can be written in the form $H=H_{A}+H_{B}+H_{AB}$, where all these terms are assumed to be time independent and where $H_{A}$ and $H_{B}$ are defined in terms of operators that only act on $H_{A}$ and $H_{B}$, respectively. In particular, $[H_{A},H_{B}]=0$ and $H_{AB}$ contains all terms that couple the subsystems *A* and *B*. In general, $[H_{A},H_{AB}]\neq 0$ and $[H_{B},H_{AB}]\neq 0$, but we expect these commutators to be relatively small since they only involve operators on the boundary between *A* and *B*. More precisely, in a *d*-dimensional lattice system with $O(N^{d})$ sites, we expect expectation values of $H_{A}$ and of $H_{B}$ to be $O(N^{d})$, while those of $H_{AB}$ and its commutators to be $O(N^{d-1})$.

This is important as we analyze the criteria in Equations (7) and (8). We first note that orthogonality is preserved under the evolution generated by $H_{B}$ alone, i.e.,

(10) $$\langle \phi_{1}|\phi_{2}\rangle =\langle \phi_{1}|e^{iH_{B}t}e^{-iH_{B}t}|\phi_{2}\rangle =0\;.$$

Now, we write the full evolution operator as $U_{t}=\exp\left[-i\left(H_{A}+H_{B}+H_{AB}\right)t/\hbar \right]$. By using the Zassenhaus formula (a relative of the Baker–Campbell–Hausdorff formula) twice, we can write

(11) $$\begin{array}{cc} U_{t} & \approx e^{-iH_{B}t/\hbar }e^{-iH_{A}t/\hbar }e^{-iH_{AB}t/\hbar }\\ \; & \times \;e^{-\frac{1}{2}\left[H_{A},H_{AB}\right]{\left(-it/\hbar \right)}^{2}}e^{-\frac{1}{2}\left[H_{B},H_{AB}\right]{\left(-it/\hbar \right)}^{2}}\;, \end{array}$$

where higher order factors involve nested commutators of $H_{B}$ and $H_{AB}$, higher powers of −it/ℏ, and increasingly small coefficients in the exponents, all of which generally tend to suppress their relative importance [42]. The factors leading to any violation of Equation (8) are thus systematically suppressed. This is in addition to the difference in scaling of $H_{A}$, $H_{B}$, and $H_{AB}$ already mentioned. An exact analysis of these factors will depend on the system.

For example, in this paper, we look in detail at the the Heisenberg spin chain model with *N* sites and periodic boundary conditions, which has Hamiltonian

(12) $$H=-J\sum_{n=1}^{N}S_{n}\cdot S_{n+1}\;,$$

where $S_{n}=\left(S_{n}^{x},S_{n}^{y},S_{n}^{z}\right)$ is the spin operator at site *n*. We take *A* to be a block of adjacent spins going from site 1 to $N_{A}$, and in this case,

(13) $$H_{AB}=-J\left(S_{N}\cdot S_{1}+S_{N_{A}}\cdot S_{N_{A}+1}\right)\;.$$

From this, we can see that $\left[H_{A},H_{AB}\right]$ and $\left[H_{B},H_{AB}\right]$ will involve terms of the form

(14) $$\begin{array}{cc} {}[S_{n-1}\cdot S_{n}, & S_{n}\cdot S_{n+1}]=\left[S_{n-1}^{i}S_{n}^{i},S_{n}^{j}S_{n+1}^{j}\right]\\ \; & =S_{n-1}^{i}S_{n+1}^{j}\left[S_{n}^{i},S_{n}^{j}\right]\\ \; & =S_{n-1}^{i}S_{n+1}^{j}i\hbar \epsilon^{ijk}S_{n}^{k}\\ \; & =i\hbar \epsilon^{ijk}S_{n-1}^{i}S_{n+1}^{j}S_{n}^{k}\\ \; & =i\hbar \left(S_{n-1}\times S_{n+1}\right)\cdot S_{n}\;, \end{array}$$

where we have used the fact that spin operators at different sites commute. Applying this, we have

(15) $$\begin{array}{cc} {}[H_{B},H_{AB}]/i\hbar & =\left(S_{N-1}\times S_{1}\right)\cdot S_{N}\\ \; & -\left(S_{N_{A}}\times S_{N_{A}+2}\right)\cdot S_{N_{A}+1}\;, \end{array}$$

and

(16) $$\begin{array}{cc} {}[H_{A},H_{AB}]/i\hbar & =\left(S_{N_{A}-1}\times S_{N_{A}+1}\right)\cdot S_{N_{A}}\\ \; & -\left(S_{N}\times S_{2}\right)\cdot S_{1}\;. \end{array}$$

We see from Equations (13), (15) and (16) that $H_{AB}$ and its commutators scale like the boundary of *A*, which in this one dimensional case, is just fixed to be two lattice points or $O(N^{0})$ (while $H_{A}$ and $H_{B}$ will generally have O(N) terms). When inserted into the Zassenhaus expansion in Equation (11), these factors may lead to some violation of Equation (8), but this will be suppressed in the limit of a large lattice. This is consistent with the Lieb–Robinson bound, which allows for small corrections [38].

**Figure 1:**
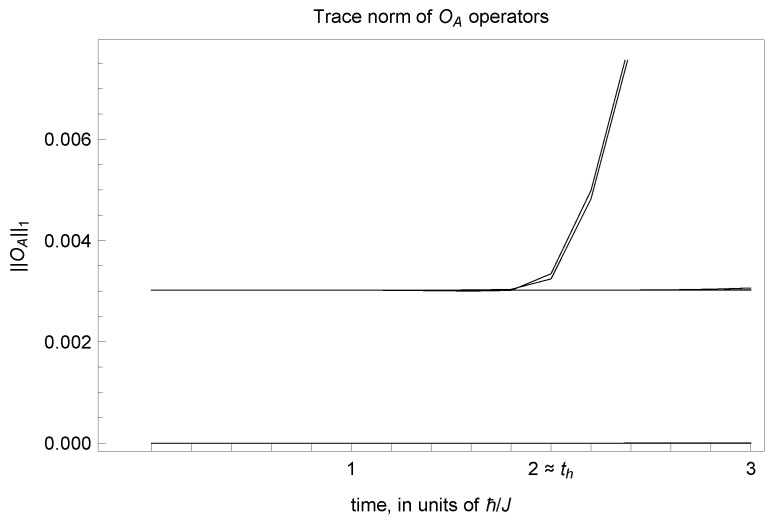
We show the evolution of the trace norm of operators $O_{A}\left(t\right)={\mathrm{tr}}_{B}|\Psi_{1}\left(t\right)\rangle \langle \Psi_{2}\left(t\right)|$ formed from equivalent states, as defined in Equation (8), in the case of the Heisenberg model discussed in detail below. Here, *A* corresponds to a single spin at site 1 and the uppermost curve comes from a pair of equivalent states with two spin flips at sites (4,5) and (5,6), respectively. That is, right outside the horizon of *A*, which is defined by $r_{h}=2$ and $t_{h}\approx 2$. This leads to its slightly larger value, compared to the other randomly chosen pairs of equivalent states, which all give smaller trace norms.

As part of this work, we generated numerical evidence that these corrections do indeed remain small in the system we consider. In particular, we considered a set operators like those appearing in Equation (8), constructed from 14 randomly chosen pairs of equivalent states in $H_{B}$, and verified that their trace norm remained small, $O(10^{-3})$ or less, up until the time horizon. We plot a sample of these data in Figure 1. In comparison, the trace distance between reduced density operators coming from inequivalent states is O(1).

### 2.2. Predictive States

Any equivalence relation on $H_{B}$ can be seen as a map from it to a quotient space $H_{B}^{\prime }$, which we will call the predictive state space. We will see below that the quotient map is a linear projection map and $H_{B}^{\prime }$ can be considered a Hilbert space itself. Here, we focus on how the quotient map affects the reduced density operator $\rho_{A}$ and its entropy, and how to interpret the results information-theoretically.

Let us consider a Hilbert space $H_{B}$ of arbitrary dimension $N_{3}$. Suppose $\left\{|\varphi_{i}\rangle \right\}$ is an orthonormal basis for a linear subspace of equivalent states, of dimension $N_{1}$. Let $N_{2}$ be the dimension of the orthogonal complement of the subspace, so that $N_{1}+N_{2}=N_{3}$. We define a linear projection operator *P* that maps all difference vectors $|\varphi_{i}\rangle -|\varphi_{j}\rangle$ to zero. This projects the subspace onto the span of the normalized vector $|\gamma \rangle =\left(1/\sqrt{N_{1}}\right)\left(\sum_{i=1}^{N_{1}}|\varphi_{i}\rangle \right)$ and may be written as $P={\mathrm{Id}}_{N_{2}}+\left|\gamma \rangle \langle \gamma \right|$.

To have a probabilistic interpretation of the states after projection, they must be normalized. In line with the idea of grouping equivalent states, we require that the probability of the predictive state equal the sum of all the probabilities in the equivalence class. A general state $|\Psi \rangle \in H=H_{A}⊗H_{B}$ may be written in a Schmidt decomposition as

(17) $$|\Psi \rangle =\sum_{i=1}^{\dim H_{A}}\sum_{j=1}^{N_{3}}a_{ij}|\psi_{i}\rangle |\phi_{j}\rangle \;.$$

The normalization of the equivalent states will have a non-trivial effect on terms of the form $a_{1}{|\psi \rangle }_{i}|\varphi_{1}\rangle +\ldots +a_{N_{1}}{|\psi \rangle }_{i}|\varphi_{N_{1}}\rangle$, i.e., terms that include equivalent states. In our prescription, these terms will be mapped to the following in the normalized state $|\Psi^{\prime }\rangle$:

(18) $$\sqrt{\sum_{j}{\left|a_{j}\right|}^{2}}\frac{\sum_{j}a_{j}}{\left|\sum_{j}a_{j}\right|}|\psi_{i}\rangle |\gamma \rangle \;,$$

where the sums are from 1 to $N_{1}$. The first factor, involving a square root, is the normalization necessary for a consistent probabilistic interpretation of the coefficients. The second factor is a unit complex number that comes from the projection by *P*. This normalizing of coefficients makes the overall map, from the normalized states in $H_{B}$ to the normalized states in $H_{B}^{\prime }$, non-linear.

In the case of additional, distinct subspaces of equivalent states, the discussion above is iterated and more coefficients will be involved. The equivalent subspaces may be directly summed into an increasing sequence and so, in linear algebra terms, form a (partial) flag for $H_{B}$. This is the same kind of structure that appears when decomposing a Hilbert space into superselection sectors [43]. The relevant projection operator *P* is then the identity on the subspace of inequivalent states plus a sum over projectors $|\gamma_{i}\rangle \langle \gamma_{i}|$ onto rays, one for each distinct subspace of equivalent states. We omit the explicit general expressions since we do not need them for our purposes here.

In the end, we are interested in a normalized state $|\Psi^{\prime }\rangle$ in the predictive state space $H_{B}^{\prime }$, and we have implicitly defined it to be P|Ψ〉 after its coefficients are normalized accordingly (18). We may then define the predictive state density operator $\rho^{\prime }=|\Psi^{\prime }\rangle \langle \Psi^{\prime }|$ and its partial traces $\rho_{B}^{\prime }={\mathrm{tr}}_{A}\rho^{\prime }$ and $\rho_{A}^{\prime }={\mathrm{tr}}_{B^{\prime }}\rho^{\prime }$. Here, ${\mathrm{tr}}_{B^{\prime }}$ means a partial trace over the the predictive state space $H_{B}^{\prime }$. We work out explicit examples of these definitions in Section 2.4. The predictive states are used to define the predictive complexity, which we examine next.

### 2.3. Predictive Complexity

We define the *predictive complexity* $C_{A}$ of *A* to be the von Neumann entropy of the predictive state density operator defined above, $\rho_{A}^{\prime }={\mathrm{tr}}_{B^{\prime }}|\Psi^{\prime }\rangle \langle \Psi^{\prime }|$,

(19) $$C_{A}=-\mathrm{tr}\left(\rho_{A}^{\prime }\log\rho_{A}^{\prime }\right)\;,$$

where $|\Psi^{\prime }\rangle$ is the state formed by implementing predictive equivalence, as defined by Equation (3), and normalized according to Equation (18). We interpret $C_{A}$ as the entropy of *A* due to tracing over *A*’s environment *B*, modulo predictively equivalent states in *B*. One could similarly define other measures of predictive complexity from other information measures of $\rho_{A}^{\prime }$, such as the Renyi entropy, negativity, etc.

We conjecture that $C_{A}\leq S_{A}=-\mathrm{tr}\left(\rho_{A}\log\rho_{A}\right)$, i.e., that the predictive complexity is bounded above by the standard entanglement entropy, with equality if no states in *B* are predictively equivalent. This is true in all cases we have examined. Similarly, in practice, one may only know that a certain subspace of states in $H_{B}$ is predictively equivalent for some interval of time. For example, states supported outside of some horizon around *A*. The true set of equivalencies may be greater, and we expect this would only decrease the resulting complexity. In that case, one computes an upper bound on the predictive complexity.

Many additional questions may be asked about the predictive complexity $C_{A}$ and its properties. We address some of these in the Discussion, but many are the subject of future research, and we turn now to an example calculation of this quantity in a model system.

### 2.4. Predictive States and Complexity in a Small System

We present a low-dimensional example where we can work out the definitions of predictive state and predictive complexity explicitly. Let us consider $H_{A}≅C^{2}$ and $H_{B}≅C^{3}$. Let ${\{|1\rangle }_{A}{,|2\rangle }_{A}\}$ be an orthonormal basis for $H_{A}$ and ${\{|1\rangle }_{B}{,|2\rangle }_{B}{,|3\rangle }_{B}\}$ be one for $H_{B}$, and suppose that ${|1\rangle }_{B}$∼${|2\rangle }_{B}$, i.e., they are found to be equivalent under Equation (3). Form the normalized sum and difference vectors $|\alpha \rangle :=\left(1/\sqrt{2}\right){(|1\rangle }_{B}-{|2\rangle }_{B})$ and $|\beta \rangle :=\left(1/\sqrt{2}\right){(|1\rangle }_{B}+{|2\rangle }_{B})$. We form the projection operator $P={|3\rangle }_{B}{\langle 3|}_{B}+\left|\beta \rangle \langle \beta \right|$ on $H_{B}$ that projects along |α〉 and onto its orthogonal complement, the image of *P*. Call that image $H_{B}^{\prime }$, in this case, a two (complex)-dimensional subspace, spanned by ${|3\rangle }_{B}$ and |β〉.

Let us look first at a general state in the 1−2 subspace of $H_{B}$, where we have $P{(a_{1}|1\rangle }_{B}+a_{2}{|2\rangle }_{B})=\frac{1}{2}\left(a_{1}+a_{2}\right){(|1\rangle }_{B}+{|2\rangle }_{B})=\frac{1}{\sqrt{2}}\left(a_{1}+a_{2}\right)|\beta \rangle$. If we normalized this state, we would end up with

(20) $$\frac{a_{1}+a_{2}}{|a_{1}+a_{2}|}|\beta \rangle \;.$$

We note that if $a_{1}+a_{2}=0$, the state is mapped to the zero vector and so lies in the kernel of *P*. Such states are effectively excluded from the final state space and are concomitant of having state vectors that are physically equivalent in some sense. The same phenomenon occurs in quantization of gauge theories, as in the BRST formalism, and we plan to elaborate on this connection in future work.

Next, take a general normalized state in H, given by

(21) $$\begin{array}{cc} |\psi \rangle & =a_{1}|11\rangle +a_{2}|12\rangle +a_{3}|13\rangle \\ & +a_{4}|21\rangle +a_{5}|22\rangle +a_{6}|23\rangle \;, \end{array}$$

where $|ij\rangle ={|i\rangle }_{A}⊗{|j\rangle }_{B}$. Under *P*, this state is mapped to the unnormalized state

(22) $$P|\psi \rangle =\frac{1}{\sqrt{2}}\left(a_{1}+a_{2}\right){|1\rangle }_{A}|\beta \rangle +a_{3}{|1\rangle }_{A}{|3\rangle }_{B}+\frac{1}{\sqrt{2}}\left(a_{4}+a_{5}\right){|2\rangle }_{A}|\beta \rangle +a_{6}{|2\rangle }_{A}{|3\rangle }_{B}\;.$$

To normalize this state, we focus on the terms that have been affected by the projection. For example, the contribution of the first two terms in Equation (21) to the norm of |ψ〉 was $|a_{1}{|}^{2}+{\left|a_{2}\right|}^{2}$. For a consistent probabilistic interpretation of the coefficients, we need that to be the contribution of the first term in the projected state. Thus, we define the normalized state

(23) $$\begin{array}{cc} |\psi^{\prime }\rangle & =\sqrt{|a_{1}{|}^{2}+{\left|a_{2}\right|}^{2}}\frac{a_{1}+a_{2}}{|a_{1}+a_{2}|}{|1\rangle }_{A}|\beta \rangle +a_{3}{|1\rangle }_{A}{|3\rangle }_{B}\\ \; & +\sqrt{|a_{4}{|}^{2}+{\left|a_{5}\right|}^{2}}\frac{a_{4}+a_{5}}{|a_{4}+a_{5}|}{|2\rangle }_{A}|\beta \rangle +a_{6}{|2\rangle }_{A}{|3\rangle }_{B}\;. \end{array}$$

We are interested in how this affects the reduced density operator $\rho_{A}={\mathrm{tr}}_{B}\left|\psi \rangle \langle \psi \right|$, whose matrix elements are defined by $\rho_{A}=\sum_{ij}\rho_{Aij}{|i\rangle }_{A}{\langle j|}_{A}$. Represented as a matrix in the given basis, the original reduced density operator is

(24) $$\begin{array}{c} \rho_{A}=\left(\begin{array}{cc} |a_{1}{|}^{2}+|a_{2}{|}^{2}+{\left|a_{3}\right|}^{2} & a_{1}a_{4}^{*}+a_{2}a_{5}^{*}+a_{3}a_{6}^{*}\\ a_{1}^{*}a_{4}+a_{2}^{*}a_{5}+a_{3}^{*}a_{6} & |a_{4}{|}^{2}+|a_{5}{|}^{2}+{\left|a_{6}\right|}^{2} \end{array}\right)\;. \end{array}$$

Under the action of the equivalence relation, we have a modified reduced density operator

(25) $$\rho_{A}^{\prime }={\mathrm{tr}}_{B^{\prime }}|\psi^{\prime }\rangle \langle \psi^{\prime }|=\langle \beta |\psi^{\prime }\rangle \langle \psi^{\prime }|\beta \rangle +\langle 3|\psi^{\prime }\rangle \langle \psi^{\prime }|3\rangle \;.$$

Represented as a matrix, the diagonal elements (and thus the trace) are unchanged, but the upper off-diagonal element is now

(26) $$\begin{array}{c} \rho_{A12}^{\prime }=a_{3}a_{6}^{*}+\sqrt{\left(\right|a_{1}{|}^{2}+|a_{2}{|}^{2})(|a_{4}{|}^{2}+|a_{5}{|}^{2})}\frac{\left(a_{1}+a_{2}\right)\left(a_{4}^{*}+a_{5}^{*}\right)}{|a_{1}+a_{2}\left|\right|a_{4}+a_{5}|}\;. \end{array}$$

The other off-diagonal element is the complex conjugate since $\rho_{A}^{\prime }$ is Hermitian. The modification of the off-diagonals affects the eigenvalues and hence the von Neumann entropy of $\rho_{A}^{\prime }$, which we identify as the predictive complexity $C_{A}$. The explicit expression for $C_{A}$ in terms of the $a_{i}$ is long and not illuminating, so we omit it.

To give a concrete example of how the predictive complexity will differ from the entanglement entropy, suppose that we have a state that is a Bell state involving entanglement between subsystem *A* and the states ${|1\rangle }_{B}$ and ${|2\rangle }_{B}$:

(27) $$|\psi \rangle =\frac{1}{\sqrt{2}}(|11\rangle +|22\rangle )\;.$$

This corresponds to $a_{1}=a_{5}=1/\sqrt{2}$ with the rest of the $a_{i}$ equal to zero. The reduced density operator, Equation (24), is diagonal, and the entanglement entropy is clearly $S_{A}=\log2$. Under the equivalence relation, ${|1\rangle }_{B}$ and ${|2\rangle }_{B}$ are in the same predictive state, and according to Equation (26), the reduced density operator $\rho_{A}^{\prime }$ picks up off-diagonal elements equal to $a_{1}a_{5}^{*}=1/2$. This changes the eigenvalues to be 1 and 0, and so we obtain $C_{A}=0$. This is consistent with the idea that in the case ${|1\rangle }_{B}∼{|2\rangle }_{B}$, the state $|\psi^{\prime }\rangle =\left(1/\sqrt{2}\right){(|1\rangle }_{A}|\beta \rangle +\left|2\rangle |\beta \rangle \right)$ factorizes and the entanglement disappears.

We have worked this out in a low-dimensional case, but the extension to an arbitrary finite-dimensional Hilbert space is relatively straightforward. In particular, in the following section, we will apply it to the N=32 site Heisenberg model.

## 3. Results: Predictive Complexity of the Heisenberg Model

### 3.1. The Heisenberg Model

It is interesting to apply these concepts to a quantum system with highly non-trivial spacetime dynamics, but that is nonetheless exactly solvable. We consider a standard spin chain from condensed matter theory, the 1D isotropic (XXX) Heisenberg model on N=32 sites, with ferromagnetic, nearest neighbor interactions and periodic boundary conditions. The Hamiltonian was given above in Equation (12). The value of *J* does not affect our analysis, so we set J=1 (equivalently, we choose time *t* to be in units of ℏ/J). Analyzing the Hilbert space of this model is greatly facilitated by the Bethe ansatz for the energy eigenstates, and we will largely follow the very helpful review article [44]. Within the two-magnon sector, we have calculated the complete time-dependent wavefunction and so can compute the entanglement entropies and predictive complexities exactly. This could also be accomplished by direct diagonalization, but we found the Bethe ansatz helpful for distinguishing contributions from scattering states and bound states.

The full Hilbert space has the dimension $2^{32}$ and has an orthogonal basis of position states $|\sigma_{1}\ldots \sigma_{N}\rangle$ and a position basis, with $\sigma_{n}=↑$ or ↓ representing spin up or down, respectively, in the *z* direction at site *n*. These position states are generally not eigenstates of the Hamiltonian, and hence evolve non-trivially with time. We will restrict our attention to the two-magnon sector, a subspace of dimension 322=496, with an orthonormal positional basis consisting of states of the form

(28) $$\begin{array}{cccc} |n_{1},n_{2}\rangle =|↑↑\cdots & ↓\cdots & \; & ↓\cdots ↑↑\rangle \;,\\ \; & n_{1} & \; & n_{2} \end{array}$$

where $1\leq n_{1}<n_{2}\leq 32$.

### 3.2. Single Site Entropy and Complexity

**Figure 2:**
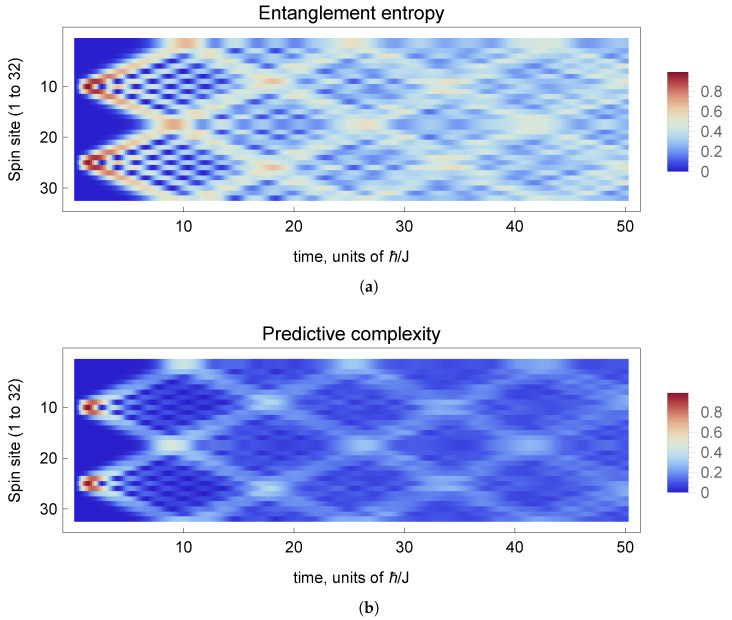
Spacetime plots of the Heisenberg spin chain after two initial spin flips at sites 10 and 25. (**a**) shows the single-site entanglement entropy, while (**b**) shows the single-site predictive complexity, with a horizon radius of two sites. The color scale indicates the value in bits (color map choice informed by [52]). Note the magnon beams and collisions appear with greater contrast in the complexity plot. We quantify this below. Calculations are performed with a time step of dt=0.2.

The entanglement entropy of subsystems of various Heisenberg models and spin chains in various states has been studied by many authors, e.g., [45,46,47], and for a review, see [48]. Here, we compare the entropy to the complexity for a subsystem *A* consisting of a single spin, in a two-magnon state with an initial condition consisting of a largely ferromagnetic state but with two spins flipped. These states evolve in time and generate entanglement that spreads to all sites, as shown in the plot of the single site entanglement entropy in Figure 2a.

The predictive complexity for a given site *j* may be defined for any choice of horizon radius, $r_{h}$. That is, the exterior region *B* is defined to be all sites except site *j*, and we apply an equivalence relation on states in *B* that only differ outside the horizon. This equivalence is straightforward to implement in the position basis. Effectively, we focus on the local environment of spin *j*, sites from $j-r_{h}$ to $j+r_{h}$ (modulo periodic boundary conditions). This is all that you need to predict the future of that spin for some duration of time due to the Lieb–Robinson bound on the speed of propagation in this system [38,49,50]. This bound is associated with light-cone-like dynamics, which can be seen in Figure 2b.

In more detail, we consider the case where *A* consists of a single spin at some arbitrary site *j* and the initial condition consists of two spins flipped. States whose only excitations (spin flips) are outside the horizon of *A* are taken to be equivalent. The horizon is determined by the horizon radius $r_{h}$, which is a free (discrete) parameter. Because the dynamics of the Heisenberg model are local and obey a Lieb–Robinson bound, the horizon distance is proportional to the predictive (time) horizon (we do not need the value of the Lieb–Robinson velocity for our purposes here, but calculating such values is an active area of research [50,51]).

The Bethe ansatz basis for the two-magnon sector is composed of states of the form

(29) $$\begin{array}{cc} |k_{1},k_{2}\rangle & =\sum_{n_{1}<n_{2}}a_{k_{1}k_{2}}\left(n_{1},n_{2}\right)\;|n_{1},n_{2}\rangle \end{array}$$

(30) $$\begin{array}{cc} \; & =A_{k_{1}k_{2}}\sum_{n_{1}<n_{2}}(e^{i(k_{1}n_{1}+k_{2}n_{2}+\frac{1}{2}\theta )}+e^{i(k_{1}n_{2}+k_{2}n_{1}-\frac{1}{2}\theta )})|n_{1},n_{2}\rangle \;, \end{array}$$

where $A_{k_{1}k_{2}}$ is a constant ensuring normalization:

(31) $$\langle k_{1},k_{2}|k_{1},k_{2}\rangle =\sum_{n_{1}<n_{2}}{\left|a_{k_{1}k_{2}}\left(n_{1},n_{2}\right)\right|}^{2}=1\;.$$

Here, $k_{1}$ and $k_{2}$ are lattice momenta and are related to the energy *E* by

(32) $$E_{k_{1}k_{2}}-E_{0}=J\left(2-\cos k_{1}-\cos k_{2}\right)\;,$$

where $E_{0}$ is the energy of the state with no spin flips. The (scattering) phase θ is related to the momenta by $2\cot\left(\theta /2\right)=\cot\left(k_{1}/2\right)-\cot\left(k_{2}/2\right)$. Translation invariance of the wave function imposes further conditions. With care and some numerical effort, a complete set of solutions may be calculated; see, e.g., [44]. The spectrum includes a large class of states that may be interpreted as two-magnon scattering states, as well as bound states with complex momenta.

As mentioned above, because of the locality of Hamiltonian, Equation (12), and the Lieb–Robinson bound, if you want to predict the evolution of some segment of the spin chain for some amount of time, you only need information about the state of the system within a horizon region around *A*. Let us take *A* to be the spin $\sigma_{j}$ at some arbitrary site *j*. For definiteness, take the horizon radius $r_{h}=2$. We can depict these various regions on the spin chain as:

(33) $$\begin{array}{c} \underset{\mathrm{outside}}{\underset{︸}{\cdots \sigma_{j-3}}}\underset{\mathrm{inside}}{\underset{︸}{\sigma_{j-2}\sigma_{j-1}\overset{A}{\overset{︷}{\sigma_{j}}}\sigma_{j+1}\sigma_{j+2}}}\underset{\mathrm{outside}}{\underset{︸}{\sigma_{j+3}\cdots }}\;. \end{array}$$

Recall that *B* is defined to be the complement of *A*, i.e., all sites except *j*.

We are restricting to the two-magnon sector, so for a given value of *j*, all states $|n_{1},n_{2}\rangle$ in $H_{B}$ can be put into three types:

(I) $n_{1}$ and $n_{2}$ are outside the horizon.
(II) Either $n_{1}$ or $n_{2}$ is inside the horizon but not both.
(III) Both $n_{1}$ and $n_{2}$ are inside the horizon.

These criteria can be phrased numerically, e.g., $n_{1}$ is inside the horizon if $|j-n_{1}\;\mathrm{mod}\;N|\leq r_{h}$, etc. All states of type I are equivalent to each other. Thus, there are NI=N−(2rh+1)2=272=351 states in this equivalence class in this case. States in class II will be equivalent according to whether their inside spin flip is the same. So, there are $2r_{h}=4$ equivalence classes, with $N_{\mathrm{II}}=N-\left(2r_{h}+1\right)=27$ states in each class. Finally, each of the 2rh2=6 states of type III are inequivalent to any other state.

As described above, we can associate these equivalence relations with a map from the original Hilbert space to the space of (normalized) predictive states; see Equation (18). In this case, we are interested in applying this equivalence relation (and ultimately calculating density operators and entanglement entropies) to dynamical, excited states, |Ψ(t)〉. In this work, we focus on states whose initial conditions are two individual spin flips. That is, states of the form:

(34) $$|\Psi \left(t\right)\rangle =e^{-iHt}|n_{1}^{\prime },n_{2}^{\prime }\rangle .$$

The evolution of these two-magnon states can be expanded in terms of the Bethe ansatz states, which form a complete basis of Hamiltonian eigenstates within the two-magnon sector:

(35) $$\begin{array}{cc} |\Psi (t)\rangle & =\sum_{k_{1},k_{2}}e^{-iE_{k_{1}k_{2}}t}|k_{1},k_{2}\rangle \langle k_{1},k_{2}|n_{1}^{\prime },n_{2}^{\prime }\rangle \;\\ \; & =\sum_{k_{1},k_{2}}e^{-iE_{k_{1}k_{2}}t}\;a_{k_{1},k_{2}}^{*}\left(n_{1}^{\prime },n_{2}^{\prime }\right)\;|k_{1},k_{2}\rangle \end{array}$$

where the energies $E_{k_{1}k_{2}}$ are given by Equation (32). It will be useful to expand the energy eigenstates $|k_{1},k_{2}\rangle$ in the position basis:

(36) $$\begin{array}{cc} |\Psi (t)\rangle & =\sum_{k_{1},k_{2}}[e^{-iE_{k_{1}k_{2}}t}\;a_{k_{1},k_{2}}^{*}\left(n_{1}^{\prime },n_{2}^{\prime }\right)\times \left(\sum_{n_{1}<n_{2}}a_{k_{1},k_{2}}\left(n_{1},n_{2}\right)|n_{1},n_{2}\rangle \right)]\;, \end{array}$$

and to define the time-dependent matrix elements

(37) $$\begin{array}{cc} b(n_{1},n_{2},t) & =\langle n_{1},n_{2}|\Psi \left(t\right)\rangle \\ \; & =\sum_{k_{1},k_{2}}[e^{-iE_{k_{1}k_{2}}t}\;a_{k_{1},k_{2}}^{*}\left(n_{1}^{\prime },n_{2}^{\prime }\right)\times \left(\sum_{n_{1}<n_{2}}a_{k_{1},k_{2}}\left(n_{1},n_{2}\right)\right)]\;, \end{array}$$

To map the state in Equation (36) to its corresponding predictive state, apply the classification into types I, II, and III, above, to the sum over $n_{1}<n_{2}$, then apply the formula in Equation (18). The result can be summarized as

(38) $$\begin{array}{cc} \; & |\Psi^{\prime }\left(t\right)\rangle =\sum_{k_{1},k_{2}}[e^{-iE_{k_{1}k_{2}}t}\;a_{k_{1},k_{2}}^{*}\left(n_{1}^{\prime },n_{2}^{\prime }\right)\times \\ \; & ((\sum_{n_{1},n_{2}\in \mathrm{out}}|a_{k_{1},k_{2}}\left(n_{1},n_{2}\right){|}^{2}{)}^{1/2}e^{i\theta_{I}}|\gamma_{I}\rangle +\\ \; & \sum_{n_{1}\in \mathrm{in}}(\sum_{n_{2}\in \mathrm{out}}|a_{k_{1},k_{2}}\left(n_{1},n_{2}\right){|}^{2}{)}^{1/2}e^{i\theta_{n_{1}}}|\gamma_{n_{1}}\rangle +\\ \; & \sum_{n_{1},n_{2}\in \mathrm{in}}a_{k_{1},k_{2}}\left(n_{1},n_{2}\right)|n_{1},n_{2}\rangle )]\;. \end{array}$$

Here, the gamma states are those used to define the linear projection, specifically $|\gamma_{I}\rangle =\left(1/\sqrt{N_{I}}\right)\sum_{n_{1},n_{2}\in \mathrm{out}}|n_{1},n_{2}\rangle$ and $|\gamma_{n_{1}}\rangle =\left(1/\sqrt{N_{\mathrm{II}}}\right)\sum_{n_{2}\in \mathrm{out}}|n_{1},n_{2}\rangle$. The phase factors are normalized sums of the amplitudes, e.g.,

(39) $$e^{i\theta_{I}}=\frac{\sum_{n_{1},n_{2}\in \mathrm{out}}a_{k_{1},k_{2}}\left(n_{1},n_{2}\right)}{\left|\sum_{n_{1},n_{2}\in \mathrm{out}}a_{k_{1},k_{2}}\left(n_{1},n_{2}\right)\right|}$$

The last three lines of (38) correspond to the classes I, II, and III, respectively.

To compute entropies, one needs the matrix elements of the density operator and reduced density operator. These are determined fairly straightforwardly from the coefficients in (38). For example, in the main part of the paper, we examined the single site entanglement entropy and predictive complexity, where the subsystem *A* consisted of a single spin, and we set the horizon radius $r_{h}=2$. The usual reduced density operator is then formed by tracing over the Hilbert spaces for all the sites except *A*. Similarly, the predictive complexity is computed from the modified reduced density operator $\rho_{A}^{\prime }$, which is obtained by tracing over the predictive state space in the complement, $H_{B}^{\prime }$. It works out that the diagonal elements of $\rho_{A}$ and $\rho_{A}^{\prime }$ are the same (as in the 5D Hilbert space example considered above), so let us turn to the off-diagonal elements.

We note, first, that the off-diagonal elements of the unmodified $\rho_{A}$ are zero, essentially because we are working within the two-magnon sector, which is preserved by time evolution. To see this, note that

(40) $$\begin{array}{cc} \langle {↓}_{j}|\rho_{A}|{↑}_{j}\rangle & =\langle {↓}_{j}|{\mathrm{Tr}}_{B}(|\Psi \rangle \langle \Psi |)|{↑}_{j}\rangle \;, \end{array}$$

and recall that |Ψ〉 is the time evolution of the two-magnon state $|n_{1}^{\prime },n_{2}^{\prime }\rangle$ (see Equation (34)). For the off-diagonal element in Equation (40) to be non-zero, one would need a term in $\sum_{B}\langle \phi_{B}|\Psi \rangle$ (where the sum is over all $|\phi_{B}\rangle \in H_{B}$) that simultaneously had a non-zero matrix element with $\langle {↓}_{j}|$ and with $\langle {↑}_{j}|$. Such a term would need to come from a state $|\phi_{B}\rangle$ that is simultaneously in the one- and two-magnon sector of $H_{B}$, which does not exist.

However, for $\rho_{A}^{\prime }$, it is possible for the off-diagonal elements to be non-zero because one- and two-magnon states can be lumped together in the same equivalence class. This allows the argument given above to be evaded. Indeed, for this particular calculation, this is the reason that the predictive complexity is different from the standard entanglement entropy at all. The off-diagonal elements can be worked out along the lines of Equation (26). Applying this, we can calculate the off-diagonal elements in terms of the wave function elements defined in Equation (37):

(41) $$\begin{array}{cc} \langle {↓}_{j}|\rho_{A}^{\prime }|{↑}_{j}\rangle & =((\sum_{n_{1},n_{2}\in \mathrm{out}}{\left|b\left(n_{1},n_{2},t\right)\right|}^{2})\\ \; & \;\;\;\;\;\;\times (\sum_{n_{2}\in \mathrm{out}}{\left|b\left(j,n_{2},t\right)\right|}^{2}){)}^{1/2}\\ \; & \times \frac{\sum_{n_{1},n_{2}\in \mathrm{out}}b\left(n_{1},n_{2},t\right)}{\left|\sum_{n_{1},n_{2}\in \mathrm{out}}b\left(n_{1},n_{2},t\right)\right|}\\ \; & \times \frac{\sum_{n_{2}\in \mathrm{out}}b\left(j,n_{2},t\right)}{\left|\sum_{n_{2}\in \mathrm{out}}b\left(j,n_{2},t\right)\right|}\;. \end{array}$$

These non-zero off-diagonal elements affect the eigenvalues of $\rho_{A}^{\prime }$ and, thus, also affect its von Neumann entropy. We identify the latter as the predictive complexity of the subsytem *A*. We present numerical calculations and analysis of this quantity for the 32-site Heisenberg model, which we obtained using the above formulas, implemented in Mathematica ver. 13.1 (code available upon reasonable request). We note that the phase factors in lines three and four of Equation (41) do not affect the eigenvalues of the two-by-two density matrix $\rho_{A}^{\prime }$, so they do not need to be calculated to just obtain the predictive complexity. This significantly speeds up the calculation.

**Figure 3:**
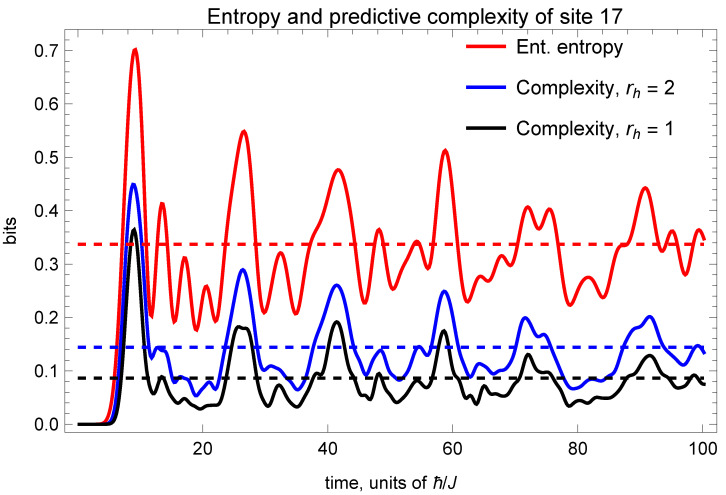
We show the evolution of the single-site entanglement entropy and predictive complexity for site 17, which sits in the middle of the two initial excited sites 10 and 25. The dashed horizontal lines indicate the equilibrium values. We note that the large local maxima in the three curves correspond to magnon collisions, and these peaks are more significant (relative to equilibrium) for the complexities than they are for the entanglement entropy. Calculations were performed using a time step of dt=0.2.

The single-site entanglement entropies and predictive complexities are shown in spacetime diagrams in Figure 2. We can see that the complexity appears to be more localized on the magnon beams and magnon collisions, while the entanglement entropy appears to show greater fluctuations throughout the spin chain. In particular, we can verify that the predictive complexity gives greater relative contrast to magnon collisions, in the sense that the local maxima associated with the collisions have a greater relative strength (compared to equilibrium) than appears with the entanglement entropy. This can be see in Figure 3, which plots the values for site 17 (midway between the initial spin flips), where the first magnon collision occurs at t≈9.0 (in units of ℏ/J). Quantitatively, the ratio of the first peak in the entanglement entropy to the equilibrium value is $S_{A}\left(t=9.0\right)/\langle S_{A}\rangle \approx 2.08$. The corresponding ratio for the complexity, taking a horizon radius of one site ($r_{h}=1$), is $C_{A}\left(t=9.0\right)/\langle C_{A}\rangle \approx 4.13$ or an increase of relative significance by a factor of almost two. Subsequent collisions are similar, though the increase is by a smaller factor.

The increased significance of the peaks, which we are interpreting as magnon collisions, can be understood from the fact that the single-site entanglement entropy is subject to contributions from entanglement between that single site and all other sites in the spin chain. The predictive complexity, on the other hand, discriminates between local and long-range entanglement. Since collisions are an inherently local phenomenon, it makes sense that the complexity would pick this up. The same reasoning can explain why fluctuations of the complexity are suppressed, compared with the entropy, as one can see in Figure 3. The standard deviation of $S_{A}\left(t\right)$ is approximately twice that of $C_{A}\left(t\right)$ (with $r_{h}=1$) at late times, after the system has thermalized. These same effects are present, to a lesser extent, for larger values of the horizon radius.

## 4. Discussion

We have presented a generalization of the reduced density operator, based on the equivalence classes that we called predictive states, and defined its entropy to be the predictive complexity. When applied to the dynamics of the Heisenberg model, this complexity highlighted dynamically significant events like magnon propagation and, especially, collisions, with an improved effective signal-to-noise ratio. This indicates that it may be an improved local order parameter for some processes, and may have applications in magnon transport [53] or quantum magnonics [54], and even more directly in recent experimental realizations of Heisenberg models [55,56].

The results presented here offer a proof of concept and should be straightforward, in principle, to generalize to other spin chains. We acknowledge that more elaborate and realistic examples would be desirable, including systems in higher dimensions, with infinite dimensional Hilbert spaces, and those described by quantum field theories. While a thorough analysis is a subject for future work, we make some initial comments here. A promising first example to consider could be the harmonic systems studied in [57,58]. Like a spin chain, such systems of locally coupled oscillators should have space and time horizons and non-trivial predictive equivalencies.

We also look forward to defining predictive complexity for quantum field theories, though this will involve some new subtleties. We aim to make contact with many powerful results, such as for entanglement entropy, Rényi entropy, and entanglement negativity in conformal field theories, particularly in dynamical situations [59]. In particular, the entanglement of two or more disjoint intervals with an environment has been extensively studied [60,61], and the notion of predictive complexity should be extendable to such cases since one can define horizons and causal diamonds for disjoint sets. One of the central tools in this domain are twist operators, which allow one to calculate powers of reduced density operators and various kinds of partial traces with relative ease. These should still be important for a calculation of predictive states and complexity in conformal field theory since they still involve partial traces outside of a given subsystem. Predictive states, though, also require the imposition of an equivalence relation outside a horizon. BRST symmetries and operators may be helpful in formulating this equivalence in field theories [62]. Predictive states may perhaps also be formulated as special kinds of boundary states in boundary conformal field theory [63]. We also note recent studies of symmetry-resolved entanglement entropy [64] and other generalized entanglement entropies [65], which would be interesting to incorporate with the concept of predictive equivalence.

We emphasize that while we have paid special attention to the scalar quantity of predictive complexity, predictive states contain far more information than this single quantity (a point also made, in a non-quantum context, in [22]). We have offered a systematic way to construct a local effective theory, in the sense of a massively reduced effective Hilbert space, $H_{B}^{\prime }$, for the environment of a given subsystem *A*, that is nonetheless sufficient for arbitrarily good predictions up to a given time horizon. In this regard, it is similar in spirit to density matrix renormalization [66], density matrix embedding [67,68], and quantum flow [69]. These methods differ from ours in that they integrally involve variational or optimization problems, and they do not involve predictive equivalence or information theoretic quantities in the same way. In any case though, it would be illuminating to identify the conceptual and computational connections between these approaches and ours.

We emphasize one way in which the predictive complexity clearly contains information distinct from the Rényi entropy, entanglement entropy, or any other quantity that does not include a horizon scale. Namely, a horizon means the predictive complexity $C_{A}$ is inherently sensitive to short-range entanglement. Furthermore, since we expect $C_{A}\leq S_{A}$, the quantity $S_{A}-C_{A}$ may be a useful new measure of long-range entanglement. Relatedly, since $C_{A}$ depends on at least two length scales, the length scale of *A* and the horizon radius $r_{h}$, we expect it to obey a variation of the usual area law for the entanglement entropy. Like the two-interval entanglement entropy (or mutual information) in conformal field theory [60], it may be sensitive to non-universal information about the spectrum of states in the theory. This and other properties of predictive states and complexity are subjects for future work.

## Data Availability

The raw data supporting the conclusions of this article will be made available by the corresponding author upon reasonable request.
